# Advances and Applications of Genome Editing in Precision Crop Breeding

**DOI:** 10.3390/plants15193057

**Published:** 2026-10-07

**Authors:** Jing Zhao, Xueyan Qian, Xiaofang Zhong, Hongwei Xun, Dongquan Guo

**Affiliations:** 1 Institute of Agricultural Biotechnology, Jilin Provincial Key Laboratory of Agricultural Biotechnology, Jilin Academy of Agricultural Sciences (Northeast Innovation Center of Agricultural Science and Technology in China), Changchun 130033, China; 2 Key Laboratory of Molecular Epigenetics of the Ministry of Education (MOE), Northeast Normal University, Changchun 130024, China

**Keywords:** genome editing, CRISPR/Cas, base editing, prime editing, crop improvement

## Abstract

Genome editing is becoming an important tool for crop improvement by enabling precise modification of genetic variation. CRISPR-Cas technologies have expanded beyond nuclease-induced mutations to include base and prime editing, transcriptional and epigenetic regulation, multiplex editing, organelle editing and targeted DNA insertion. These advances support the development of agronomically valuable alleles and extend crop improvement beyond conventional selection, recombination and random mutagenesis. Applications now include plant architecture, yield-related traits, resistance to biotic and abiotic stresses, seed composition and nutritional quality, and extend to de novo domestication, haploid induction and hybrid breeding. Current research is increasingly moving from single-gene modification toward the engineering of promoters, *cis*-regulatory elements, quantitative alleles and multiple-gene editing, particularly in polyploid crops. Wider breeding applications will depend on efficient delivery and regeneration, recovery of transgene-free and heritable edited germplasm, reliable targeted insertion and validation across relevant environments. Integration with pangenomics, multi-omics, phenomics, artificial intelligence and predictive breeding may further improve target identification, allele design and prediction of editing outcomes, thereby supporting the development of more precise and efficient crop-breeding strategies.

## 1. Introduction

Global crop production is increasingly constrained by rising food demand, climate change and limited agricultural resources [1]. Population growth and changing dietary patterns continue to increase the demand for food and agricultural products, while extreme weather, biotic stresses and soil degradation undermine yield stability [1]. Crop improvement is therefore no longer driven by yield alone, but increasingly by the need to combine high productivity with quality, stress resilience and efficient resource use [1,2]. New breeding approaches are needed to accelerate genetic gain and enable more precise trait improvement under diverse production conditions [1,2]. Conventional breeding, induced mutagenesis and transgenic approaches have produced many successful cultivars, but each strategy has limitations when breeding objectives require precise modification of endogenous loci or coordinated optimization of multiple traits [1,2,3]. Selection from standing variation and recombination remains powerful, but it depends on available alleles and frequently requires long crossing and selection cycles [1]. Random mutagenesis increases genetic diversity, but favorable variants must be recovered from a large background of unrelated mutations [1]. Transgenic technology broadens the accessible gene pool, but trait performance can be affected by insertion position, transgene expression stability and regulatory constraints [1,2,3].

Genome editing offers a way to address these constraints by enabling targeted modification of endogenous genomic sequences [1,2]. Since clustered regularly interspaced short palindromic repeats (CRISPR)-associated (Cas) systems were successfully applied in plants, nuclease-mediated editing has become a core approach for targeted gene knockout and functional validation in crops [2,3]. Base and prime editors subsequently enabled more precise nucleotide-level sequence rewriting without relying on conventional double-strand break (DSB)-mediated repair [4,5,6]. CRISPR-mediated transcriptional or epigenetic regulation, organelle editing and large-fragment DNA engineering have further expanded genome editing from coding-sequence disruption to programmable regulation and structural genome engineering [7,8,9,10]. These advances have expanded editable targets from individual coding genes to regulatory regions and larger genomic structures, improving the efficiency of functional gene characterization and trait improvement [2,3]. Genome editing is thus increasingly viewed not only as a tool for mutant generation, but also as a genome-editing platform for designed genetic variation and precision crop breeding [1,2,3]. The expansion of the genome-editing toolbox has also influenced how breeding targets are selected and modified [2,3]. Precision crop breeding integrates functionally characterized targets with deliberate allele selection or modification, recovery of heritable variation in breeding-relevant germplasm, and evaluation of agronomic performance across appropriate genetic backgrounds and environments [11,12,13]. Nuclease-mediated editing is well suited for generating loss-of-function alleles, whereas base editing and prime editing allow specific nucleotide variants and favorable alleles to be rewritten with greater precision [5,6,14,15]. Promoter and *cis*-regulatory element editing has been used to generate quantitative trait variation and tune pleiotropic genes without complete loss of function [16,17,18,19,20]. The growing capacity to generate loss-of-function, sequence-defined and quantitative regulatory alleles shifts crop improvement toward deliberate allele design rather than screening existing variation alone [2,3]. In yield-related traits, regulatory editing has improved panicle or kernel traits in rice and maize [17,18]. For resistance and quality traits, editing of *SWEET* promoters, *RBL1*, *Wx/SBEIIb* and *Coq1* illustrates how target choice can be matched to trait mechanism [21,22,23,24,25]. Breeding-system innovations such as HI-Edit and synthetic apomixis further extend genome editing from allele creation to genotype fixation [26,27]. Marker-assisted selection and genomic selection primarily exploit segregating variation already present in breeding populations, whereas genome editing provides a complementary route for recreating or modifying defined alleles when favorable variation is rare, absent from elite germplasm or difficult to combine through crossing [2,11,28]. Functional-genomics editing can establish gene or variant function, whereas breeding application additionally requires stable inheritance, recovery in breeding-relevant germplasm and reproducible agronomic performance beyond the initial molecular phenotype [1,2,11].

In polyploid crops and for complex quantitative traits, the breeding value of edited alleles is often influenced by gene dosage, genetic background and genotype-by-environment interactions [2,3,29,30]. Recent advances in pangenomics, multi-omics, high-throughput phenomics and artificial intelligence (AI) have further expanded the scope of genome editing in crop improvement [29,30]. Pangenomic and population genomic studies provide broader genetic information for identifying candidate genes, regulatory elements and favorable alleles, while revealing presence/absence variation, structural variants and cultivar-specific polymorphisms that may be missed by a single reference genome [29,31,32]. Pangenome-resolved variation can further support allele selection and guide-RNA design by distinguishing conserved target sequences from genotype-specific variation across genetically diverse germplasm [31,32]. For example, a rice pangenome constructed from 66 diverse cultivated and wild accessions identified approximately 23 million sequence variants and extensive presence/absence variation, expanding the set of candidate alleles beyond a single reference genome [33]. In tomato, graph-pangenome analysis captured structural and haplotypic variation associated with breeding-relevant traits [32]. In maize, CRISPR/Cas9 engineering of a 75.5 Mb pericentric inversion in an elite inbred line illustrates the potential of genome editing to manipulate large chromosomal structures relevant to recombination and breeding [34]. Multi-omics and high-throughput phenomic data help connect genotype, molecular mechanism and field performance, improving the reliability of target selection and editing strategy design for complex traits [12,29,35]. In parallel, artificial intelligence is increasingly used in guide RNA design and editing-outcome prediction [30,36,37]. Protein-structure prediction and generative protein design may also support future editor optimization and genotype-by-environment analysis [38,39,40,41]. Together with precise editing tools and breeding evaluation systems, these advances are promoting the transition of crop breeding from empirical selection toward more precise and design-oriented improvement, providing new opportunities for developing high-yielding, high-quality, stress-resilient and resource-efficient crop varieties [1,2,3,30]. Practical precision breeding therefore links target discovery and allele design with genotype-compatible delivery, recovery of heritable edited germplasm and multi-environment evaluation, so molecular editing efficiency alone is insufficient to judge breeding value [2,3,11].

Although recent studies have described genome-editing platforms, agricultural applications, base-editing technologies and AI-assisted breeding [2,3,4,30], the translation of genome editing into practical crop improvement increasingly requires closer integration of molecular editing with breeding processes. From a breeding perspective, the value of an editing strategy is determined not only by its molecular precision, but also by the feasibility of generating the desired allele in target genotypes, recovering heritable edited germplasm, and obtaining reproducible agronomic performance. Crop genome editing can therefore be considered as an integrated process linking target discovery and allele design, editor selection, genotype-compatible delivery and regeneration, molecular characterization of edited events, and phenotypic evaluation across relevant environments. These considerations are particularly important for quantitative traits and polyploid crops, in which gene dosage, genetic background, pleiotropic effects and genotype-by-environment interactions can influence the phenotypic consequences of editing. Pangenomics, multi-omics and phenomics provide complementary information for target identification, allele selection and functional characterization, while artificial intelligence can assist guide design, editing-outcome prediction and experimental prioritization. Integration of these approaches provides a broader basis for translating targeted genome modification into stable and agronomically useful crop improvement. Accordingly, this review considers genome-editing technologies and their applications within this breeding-oriented continuum, from target and allele design to edited-germplasm recovery and agronomic validation.

## 2. Development of Crop Genome-Editing Platforms and Technological Foundations for Genetic Improvement

### 2.1. CRISPR/Cas Nuclease Editing and Targeted Modification of Plant Genomes

**Figure 1:**
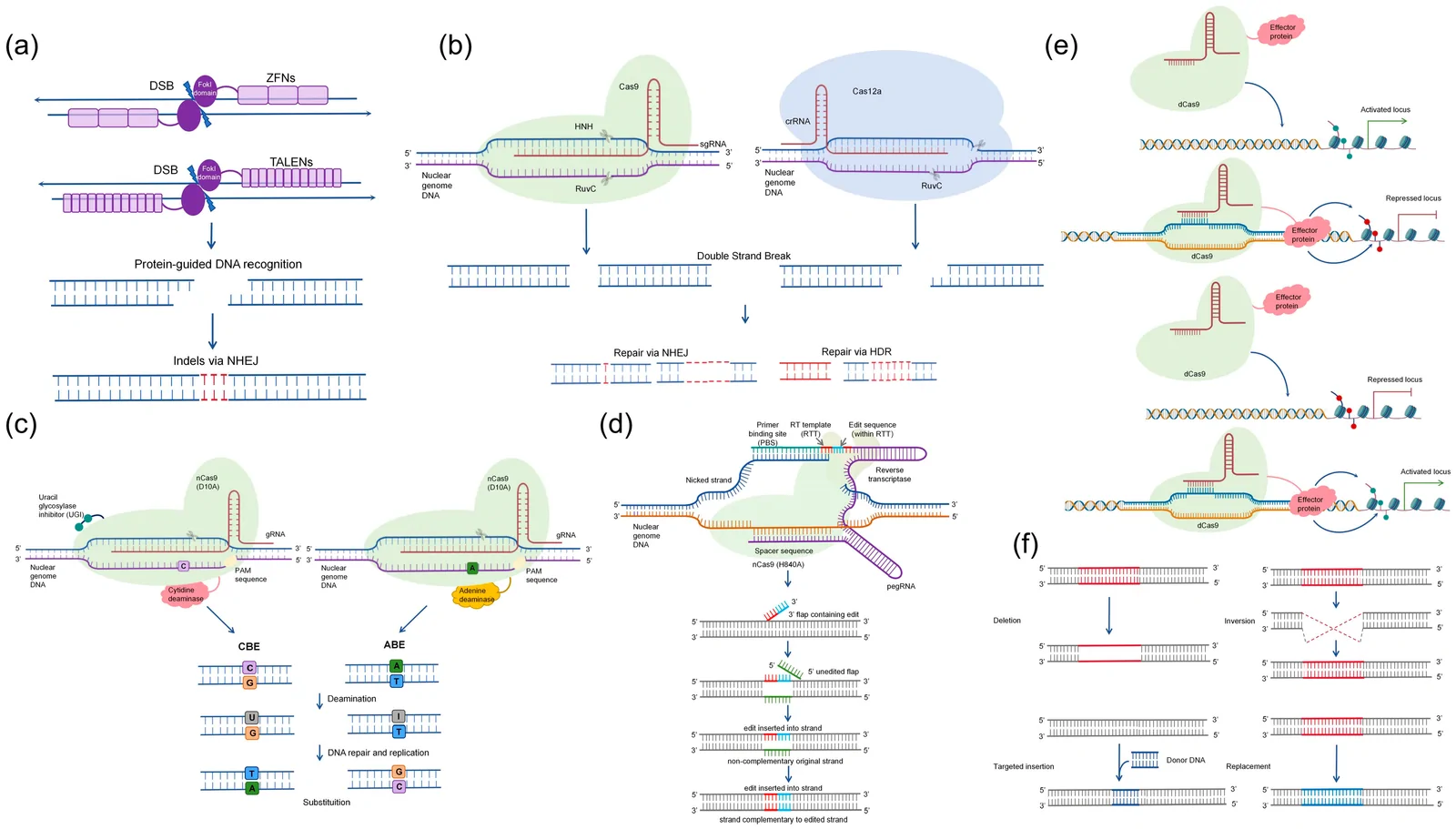
Major genome-engineering technologies. (**a**) Zinc-finger nucleases (ZFNs) and transcription activator-like effector nucleases (TALENs) use engineered protein-based DNA-binding domains to recognize paired target sites. Dimerization of the FokI nuclease domains generates a DNA double-strand break (DSB), which is frequently repaired through non-homologous end joining (NHEJ), resulting in small insertions or deletions. (**b**) CRISPR-Cas9 and CRISPR-Cas12a are directed by a single-guide RNA (sgRNA) and a CRISPR RNA (crRNA), respectively, to complementary genomic sequences. Cas9 typically generates a near-blunt DSB, whereas Cas12a produces a staggered DSB with 5′ overhangs. The resulting breaks can be repaired through NHEJ or homology-directed repair (HDR). (**c**) Base editing systems. Canonical cytosine base editors (CBEs) and adenine base editors (ABEs) couple nCas9 (D10A) to a cytidine deaminase or an engineered adenine deaminase, respectively, enabling C·G-to-T·A and A·T-to-G·C conversions without generating DSBs. The illustrated CBE also contains a uracil DNA glycosylase inhibitor (UGI). (**d**) Prime editing. A canonical prime editor comprises nCas9(H840A) fused to an engineered reverse transcriptase and a prime-editing guide RNA (pegRNA). The pegRNA specifies the genomic target and contains a primer-binding site (PBS) and a reverse-transcription template (RTT) that encodes the intended edit. (**e**) CRISPR-mediated transcriptional regulation and epigenome editing. Catalytically inactive Cas9 (dCas9) directs transcriptional regulators or epigenetic effector proteins to defined genomic loci, enabling targeted gene activation or repression and locus-specific DNA or histone modification without DNA cleavage. (**f**) Large-scale and precise genome engineering. Paired DSBs can generate large genomic deletions and inversions, whereas site-specific cleavage combined with donor-template-assisted DNA repair can mediate targeted sequence insertion and replacement.

Genome editing has reshaped the generation and use of genetic variation in crop improvement, enabling a shift from random mutagenesis and phenotype-based selection to targeted genome modification and allele-informed breeding [1,2]. Before the widespread adoption of CRISPR/Cas systems, zinc-finger nucleases (ZFNs) and transcription activator-like effector nucleases (TALENs) provided important programmable nuclease platforms for plant genome modification [42,43,44]. Both systems use engineered DNA-binding modules to recruit the FokI nuclease domain to selected genomic loci, where FokI dimerization induces DNA double-strand breaks (DSBs) [42,43,44]. ZFNs recognize DNA through arrays of zinc-finger domains, whereas TALENs achieve single-nucleotide recognition through repeat-variable diresidues within tandem TALE repeats [42,43,44] (Table 1) (Figure 1a). However, because both rely on protein-mediated DNA recognition, each new target generally requires redesign and assembly of DNA-binding proteins, limiting their scalability for multiplex editing, high-throughput functional analysis and complex trait improvement [42,43,44].

CRISPR/Cas systems shifted plant genome editing from protein-engineered nucleases to RNA-guided target recognition [9,42,43,45] (Table 1). In the CRISPR/Cas9 system, target selection is determined by guide RNA complementarity to the genomic protospacer and recognition of an adjacent PAM, allowing new targets to be programmed simply by changing the guide sequence [9,42,43,45]. Guide selection based on PAM compatibility and sequence complementarity is increasingly assisted by computational tools. CRISPR-P 2.0 (http://crispr.hzau.edu.cn/CRISPR2/ (accessed on 24 August 2026)) was developed specifically for plant genomes to identify candidate sgRNAs and assess potential off-target sites, while CRISPOR (https://crispror.labetter.fun/crispor.py (accessed on 24 August 2026)) and CHOPCHOP (https://chopchop.cbu.uib.no/ (accessed on 24 August 2026)) provide complementary functions for guide selection, ranking and target-site evaluation [46,47,48]. PAM binding and RNA–DNA hybridization promote R-loop formation and activate the HNH (Histidine-Asparagine-Histidine) and RuvC-like (related to the bacterial RuvC Holliday-junction resolvase) nuclease domains, which cleave the guide-complementary and non-complementary DNA strands, respectively, to generate a DSB [42,43,45]. In plants, these breaks are repaired mainly by non-homologous end joining (NHEJ), often producing indels that disrupt coding sequences and generate loss-of-function alleles [6,42,43,45]. Although donor-template-mediated homology-directed repair (HDR) can support precise replacement or insertion, its low efficiency in plants has limited routine knock-in and gene-replacement applications [6,42,43] (Figure 1b).

CRISPR/Cas9-mediated knockout was established early in major crops such as rice and wheat and has become a widely used tool for plant functional genomics and mutant development [1,49]. By disrupting susceptibility genes, negative regulators of quality traits and developmental regulators, this approach has supported applications in disease resistance, quality improvement, plant architecture optimization and flowering-time control [1,2]. In soybean, dual editing of *GmFAD2-1A*/*GmFAD2-1B* shifted fatty acid composition toward high oleic acid and low linoleic and linolenic acids [50]. For photoperiod and architecture-related traits, mutagenesis of *GmFT2a*/*GmFT5a* delayed flowering and affected latitudinal adaptation, while *E1*, a legume-specific flowering repressor, regulates flowering time, maturity and yield-related traits through *GmFT2a*/*GmFT5a* and related flowering pathways [49]. Editing the nuclear localization signal regions of *E1*/*E1Lb* generated allelic variants with graded functional effects, allowing modulation of photoperiod sensitivity and ecological adaptation [51]. *GmEID1* links *E3*/*E4* light signaling with the circadian Evening Complex and regulates flowering and yield formation through the *J*–*E1* module, making it a promising target for improving latitudinal adaptation [52]. Editing of *GmLHY* family genes also altered plant height and internode elongation [53]. Given soybean’s paleopolyploid origin and extensive gene duplication, multiplex editing is particularly valuable for overcoming functional redundancy, dissecting complex agronomic traits and facilitating molecular breeding [54,55].

CRISPR nuclease development is now increasingly focused on expanding target accessibility, enhancing specificity and improving delivery compatibility [2,3]. The canonical SpCas9 recognizes an NGG PAM, which restricts target selection, whereas Cas9 orthologues and engineered variants have broadened the editable sequence space and provided more flexible positioning modules for base editing, prime editing and transcriptional regulation [2,3,42,43]. Beyond Cas9, type V Cas12a nucleases have also been widely used in plant editing [3,43]. Their preference for T-rich PAMs, generation of staggered DSBs distal to the PAM and intrinsic crRNA-array processing capacity make them suitable for multiplex editing [3,43] (Figure 1b). More recently, TnpB and related compact RNA-guided nucleases have further expanded the CRISPR toolbox, with their small size offering potential advantages for viral-vector and RNP-based delivery [56,57,58,59] (Table 1). Several plant studies have demonstrated TnpB-mediated mutagenesis in *Arabidopsis*, medicinal plants and other plant systems [58,60,61]. Overall, continued nuclease diversification provides a foundation for plant genome editing platforms that are smaller, more specific and more broadly adaptable [2,3,9,43]. Compared with Cas9 and Cas12a, TnpB remains at an earlier stage of crop validation, and evidence for stable breeding deployment is still limited [55,56,57,58,59,60,61].

These nuclease platforms differ in target-site requirements, cleavage patterns, multiplexing capacity, delivery compatibility and the extent of validation in crop systems. SpCas9 remains the most extensively used platform for targeted mutagenesis in plants, whereas Cas12a expands target-site accessibility through its preference for T-rich PAMs and facilitates multiplex editing through intrinsic crRNA-array processing. Compact RNA-guided nucleases such as TnpB may provide additional flexibility for delivery because of their smaller size, but their editing performance and heritable application have so far been evaluated in fewer plant and crop systems [2,3,9,43,56,57,58,59,60,61]. Accordingly, nuclease selection should consider target-sequence constraints, multiplexing requirements, delivery strategy, crop genotype and the recovery of stable edited plants rather than editing efficiency alone.

### 2.2. Base Editing for Precise Nucleotide Substitution

Conventional CRISPR-Cas nuclease editing relies on DSB induction and NHEJ-mediated repair, which in plants usually generates small, unpredictable indels rather than defined base substitutions [6,45]. Base editing (BE) overcomes this limitation by coupling a catalytically impaired Cas protein with a nucleotide deaminase, enabling programmable base conversion without DSBs or exogenous donor DNA [4,6,14,42,43] (Table 1). Cytosine base editors (CBEs), typically composed of nCas9, a cytidine deaminase and a uracil DNA glycosylase inhibitor, convert cytosine to uracil within the exposed single-stranded DNA region, which is then resolved through replication or repair as a C:G-to-T:A transition [4,14,42] (Table 1). Adenine base editors (ABEs) use evolved TadA-derived adenine deaminases to convert adenine to inosine, which is read as guanine, thereby producing A:T-to-G:C substitutions [4,42,62] (Table 1) (Figure 1c).

Plant base editing has progressed from proof-of-concept demonstrations to a versatile toolkit with improved activity, target range and editing purity [4,6,14,42,62]. CBEs were first established in rice, wheat and maize for donor-free C-to-T substitutions, while Target-AID demonstrated single-base mutagenesis and herbicide-tolerance engineering in rice and tomato [4,14,42]. ABEs further expanded the editable mutation spectrum by enabling A:T-to-G:C conversion in plant genomes [4,62]. Subsequent advances have focused on deaminase mining, Cas variant engineering, editing-window optimization and suppression of unintended edits [6]. APOBEC3A-based CBEs enhanced editing efficiency and sequence compatibility in plants, rationally engineered cytidine deaminases improved precision, and TadA7.10, TadA8e, TadA9 and related ABE variants provided more efficient A-to-G editing platforms [4,6,42].

In crop improvement, the major value of base editing lies in creating defined allelic variation rather than disrupting genes [4,6,42]. Many agronomic traits are shaped by natural SNPs, amino acid substitutions, splice-site variants or regulatory polymorphisms [6]. Base editors can recreate favorable alleles, modify herbicide target proteins, alter splice sites and fine-tune promoter elements, supporting allelic series construction, quantitative trait dissection and molecular design breeding [4,6,42,43]. In soybean, base editing is still developing, but CBEs have been used for targeted substitutions in loci such as *GmFT2a* and *GmFT4*, and mini-Sdd7 has improved cytosine editing efficiency [49,55]. Given the numerous functional nucleotide variants underlying soybean oil composition, protein content, stress tolerance, disease resistance and photoperiod adaptation, base editing offers a promising strategy for allele-level trait improvement [4,6,7,55]. The TALE-based CyDENT system has also enabled base editing in nuclear, mitochondrial and chloroplast genomes, indicating that non-CRISPR targeting platforms may complement CRISPR editors in organellar or otherwise constrained editing contexts [7].

Base-editing design depends on the position of the desired nucleotide within the activity window of a compatible editor. BE-Designer (http://www.rgenome.net/cas-designer/ (accessed on 24 August 2026)) identifies candidate sgRNAs, editable nucleotides within defined editing windows and potential off-target sites [63]. Targetability remains constrained by PAM availability, editing-window position and bystander substrates, while broader activity windows can increase product heterogeneity [4,6,42,43]. Low-frequency indels and unintended DNA or RNA deamination remain editor-dependent risks [4,42]. Potential off-target editing also requires consideration, particularly because editor architecture, guide sequence and genomic context can influence editing specificity [4,6,42,43]. Narrower or more selective editing windows may reduce bystander substitutions and improve product purity, although this can also restrict targetable sequence space. For breeding, recovery of the intended sequence-defined allele with acceptable product purity is more informative than nominal conversion efficiency alone [4,42].

### 2.3. Prime Editing for Programmable Sequence Rewriting in Plant Genomes

Base editing enables efficient single-base conversion, but its editing spectrum is limited by deaminase chemistry and the activity window of each editor [4,5,6,64,65]. As a result, base editors cannot generate all nucleotide substitutions or readily produce precise small insertions, deletions or replacements [5,6,64,65]. Prime editing (PE) addresses this limitation by encoding the desired edit in a prime editing guide RNA (pegRNA), which combines target recognition and edit specification in a single molecule [5,43,64,65] (Table 1). A canonical PE system uses an nCas9(H840A)–reverse transcriptase fusion and a pegRNA containing a spacer, primer binding site (PBS) and reverse transcription template (RTT) [5]. PlantPegDesigner (http://www.plantgenomeediting.net (accessed on 24 August 2026)) provides an experimentally informed platform for optimizing plant pegRNA design, including spacer, PBS and RT-template configurations, and can also support paired-pegRNA strategies [66]. After nicking the non-target strand, the PBS anneals to the nicked DNA end and initiates reverse transcription, allowing the RTT-encoded sequence to be copied into a 3′ flap and incorporated through flap resolution and DNA repair [5,64,65] (Figure 1d). In plants, prime editing has been validated in rice and wheat for point mutations, small insertions and deletions, supporting its use as a programmable sequence-rewriting tool [15,64,65]. However, plant PE is generally less efficient and more variable than CBE and ABE systems, with outcomes affected by pegRNA architecture, PBS and RTT length, target-site context, reverse transcriptase activity, DNA repair status and transformation system [6,64,65]. Conventional PE2-like systems rely on the basic nCas9–reverse-transcriptase/pegRNA configuration, whereas improved plant systems combine evolved editor proteins with optimized or stabilized pegRNAs, repair modulation, additional nicking or replicon-based expression [64,65,67]. Studies have shown that the ultra-efficient prime editing system UtPE, developed in tomato, combines evolved PE6 variants, an optimized aepegRNA and a geminiviral replicon, with an NC RNA chaperone incorporated in selected configurations. Across multiple target sites, UtPE increased desired editing efficiencies by 3.39- to 8.89-fold relative to a PE2max-based system and generated heritable editing events, indicating that coordinated optimization of editor proteins, pegRNA architecture and delivery modules can improve PE efficiency and alleviate locus dependence in dicot plants [67]. Improvements have therefore focused on optimizing reverse transcriptase components, stabilizing pegRNAs, refining PBS/RTT design, introducing silent mutations to prevent re-editing, modulating DNA repair and enriching edited cells [6,64,65]. Paired pegRNA strategies further extend PE capacity by improving point mutation efficiency and enabling larger deletions, replacements and insertions [15]. Systems such as GRAND, twinPE and PRIME-Del show that PE-derived platforms are moving beyond small single-site edits toward more complex sequence deletion, replacement, insertion and inversion [8,15,64,65,68]. Studies have further shown that template-jumping prime editing (TJ-PE), when combined with the engineered reverse transcriptase variant rPE14e4, enabled precise isogenic sequence replacement of up to 250 bp in stable rice lines and the targeted rewriting of a 174 bp coding region of *xa10*. The study further showed that disrupting unintended microhomology between the PBS and RTT reduced deletion by-products and improved editing precision, providing an additional consideration for pegRNA design and optimization [69]. Despite these improvements, crop PE remains strongly dependent on pegRNA design, target locus, genotype, delivery and regeneration, and routine recovery of stable heritable edits remains a major constraint on breeding-scale deployment [6,64,65].

For crop improvement, the main strength of prime editing lies in creating predefined alleles [5,6,64,65]. Prime-editing libraries have enabled in planta screening of amino acid variants associated with herbicide resistance, illustrating their value for generating functional alleles that are difficult to obtain by conventional mutagenesis or knockout approaches [70]. In soybean, genes related to oil composition, flowering adaptation, sugar transport and disease resistance, including *GmFAD*, *GmFT*, *E1*, *GmSWEET* and *GmMLO* family members, contain functional sites suitable for precise editing [49,50,51,55]. Nevertheless, stable PE application in soybean remains constrained by large vector size, complex pegRNA design, relatively low efficiency and the time required to recover heritable edited events [55]. Improving PE efficiency across soybean genotypes is therefore essential before its routine use in breeding pipelines [55].

### 2.4. Large-Fragment Chromosomal Manipulation and Structural Genome Engineering

Genomic, pangenomic and three-dimensional genome studies have shown that agronomic traits are shaped not only by single-nucleotide variants or single-gene effects, but also by structural variation, gene clusters, distal regulatory elements and chromosome organization [29]. Large-fragment chromosomal manipulation therefore extends genome editing from nucleotide-level modification to structural genome engineering [8,29,42,43,68] (Table 1). In this strategy, gRNAs are designed at the boundaries of a target region, and Cas nucleases introduce two or more DSBs [8,68]. Repair through NHEJ, microhomology-mediated end joining, HDR or site-specific recombination can generate large deletions, inversions, duplications, translocations or targeted integrations [8,68] (Figure 1f). Compared with single-gene knockout, such approaches directly reshape genome architecture and can be used to remove unfavorable linkage blocks, rearrange chromosomal regions, regulate gene-cluster expression or integrate multiple functional elements in a modular manner [8,42,43,68].

In plant chromosome engineering, paired gRNAs are commonly used to induce two site-specific DSBs, after which DNA ends are mainly rejoined by NHEJ to produce deletions, inversions or translocations [8,42,43,68]. In *Arabidopsis*, CRISPR/Cas-mediated chromosome fusion has enabled chromosome-number remodeling and provided a system for studying chromosome evolution and structural stability [8,68]. More recently, dual-pegRNA strategies have improved the precision of large-fragment deletion and inversion, allowing kilobase- to megabase-scale structural editing in both monocots and dicots [8,68]. Prime-editing-assisted and recombinase-based strategies have expanded the precision and controllability of large-fragment genome engineering [8,68]. PrimeRoot further combines prime editing with site-specific recombination by introduc-ing recombinase recognition sites at defined genomic positions before large-fragment inte-gration, thereby improving the controllability of targeted DNA insertion [68]. These advances suggest that plant chromosome engineering is moving from structural-variant induction toward rational genome-structure design [8,42,43,68].

Transposon-related systems offer another route for targeted large-fragment insertion [8,42,43,68]. Although conventional transposons have high cargo capacity, their site specificity is limited [8,42,43,68]. Transposase-assisted target-site integration (TATSI) couples transposase activity with Cas9 or Cas12a targeting modules and has achieved targeted integration of an 8.6 kb DNA fragment in *Arabidopsis*, providing a promising strategy for plant gene knock-in [8,42,43,68]. For crop improvement, large-fragment manipulation may be used to modify long-range regulatory elements, promoter regions or three-dimensional chromatin architecture to fine-tune gene expression; integrate multiple functional elements or metabolic modules into genomic safe harbors for trait stacking and synthetic biology breeding; and generate inversions, translocations or segmental deletions to break unfavorable linkage, reshape recombination landscapes or stabilize favorable allele combinations [8,42,43,68]. However, its practical application still requires efficient delivery, robust regeneration, accurate structural-variant detection and stable inheritance, and remains technically challenging in most crops [8,42,43,68].

### 2.5. CRISPR-Mediated Transcriptional Regulation, Epigenome Editing and RNA-Targeted Regulation

The functional reach of CRISPR systems has expanded from nuclease-mediated genome modification to programmable control of gene and transcript activity [9,10,71,72]. This shift is largely enabled by nuclease-inactive Cas proteins, particularly dCas9, in which the HNH and RuvC domains are inactivated [72]. Although dCas9 no longer induces DSBs, it retains sgRNA-guided DNA binding and can serve as a programmable scaffold for transcriptional regulators, epigenetic modifiers and other effector proteins [72]. Thus, CRISPR targeting can modulate gene activity without changing DNA sequence, which is especially useful for dosage-sensitive genes, redundant gene families and essential genes that are not suitable for complete knockout [2,3,72].

CRISPR activation (CRISPRa) and CRISPR interference (CRISPRi) are representative dCas9-based expression-regulation systems [72,73,74]. (Table 1) CRISPRa commonly uses dCas9 fused to activation domains such as VP64, VPR or TV, or recruits activators through MS2 or SunTag systems, whereas CRISPRi represses transcription by blocking initiation or elongation or by recruiting repressive and chromatin-silencing factors [72]. Early plant CRISPR toolkits established the feasibility of dCas9-mediated transcriptional regulation, and CRISPR-Act3.0 further improved effector recruitment for activation of multiple endogenous genes [72,75]. CRISPR-Combo systems further integrate genome editing with transcriptional activation in plant breeding contexts [73,74]. Compared with knockout, CRISPRa/i regulates expression without disrupting gene structure, although its performance remains influenced by sgRNA position, promoter architecture, local chromatin state and expression system [72,73,76,77].

Epigenome editing integrates programmable DNA-targeting modules with effectors involved in DNA methylation or demethylation, histone modification, and other chromatin-regulatory processes [9,10,71,78] (Table 1). In CRISPR-based platforms, nuclease-dead Cas9 (dCas9) is typically coupled, either directly or through multicomponent recruitment systems, to DNA methyltransferases, demethylases, histone acetyltransferases, histone deacetylases, or histone methylation regulators, thereby enabling locus-specific modulation of epigenetic marks, chromatin accessibility, and transcription [9,10,71,78] (Figure 1e). Because the underlying DNA sequence generally remains unchanged, epigenome editing provides a powerful approach for establishing causal relationships among epigenetic marks, *cis*-regulatory elements, and developmental or stress-responsive gene-expression programs [10,71,78]. In *Arabidopsis*, distinct dCas9–SunTag configurations have been used for transcriptional activation and targeted DNA methylation. Recruitment of the transcriptional activator VP64 enabled activation of the *FWA* locus, whereas recruitment of the *Nicotiana tabacum* DRM methyltransferase catalytic domain (NtDRMcd) directed DNA methylation to the *FWA* and *SUPERMAN* promoters [78]. The applicability of targeted epigenome modification to crop improvement has also been demonstrated in cassava. An engineered zinc-finger–DMS3 fusion directed DNA methylation to the TAL20 effector-binding element within the *MeSWEET10a* promoter, thereby suppressing TAL20-dependent *MeSWEET10a* activation and reducing cassava bacterial blight symptoms. These findings demonstrate the potential of targeted DNA methylation for improving crop disease resistance without altering the target DNA sequence [79]. The CRISPRepi database further integrates RNA-seq, ChIP-seq, bisulfite-seq, and ATAC-seq datasets to support guide RNA design, evaluation of editing outcomes, and assessment of potential off-target effects [10,71,78]. Heritable crop-breeding evidence for epigenome editing remains limited, with persistence and inheritance depending on locus, epigenetic mark and editor system [9,10,78,79].

RNA-targeted CRISPR systems extend programmable regulation from the genome to the transcriptome [9,80,81] (Table 1). Cas13 nucleases recognize RNA targets through crRNA-guided pairing and cleave transcripts via higher eukaryotes and prokaryotes nucleotide-binding (HEPN) domains, whereas catalytically inactive Cas13 variants can be fused to RNA-modifying enzymes for transcript-level base editing [9,80,81]. Because RNA regulation does not alter genomic DNA, its effects can be reversible and temporally controllable, offering advantages for essential gene analysis, viral RNA suppression, transient stress-response regulation and transcript dosage control [9,80,81]. In plants, multiple Cas13 orthologues have been evaluated for RNA knockdown, including silencing of endogenous *GhCLA* and *GhPGF* transcripts in cotton and reduction in *Tobacco mosaic virus* (TMV) accumulation in tobacco [59]. These studies demonstrate the utility of Cas13-based systems for functional genomics and antiviral applications, but transcript cleavage and RNA modification should be distinguished from stable genome editing for crop breeding [3,81]. Practical breeding applications will depend on efficient delivery, appropriate expression in target tissues and reproducible phenotypic effects. RNA-targeted systems therefore remain largely experimental or proof-of-concept approaches in plants, and evidence for their stable application in crop breeding remains limited [9,81].

**Table 1:** Major genome-editing technologies, molecular components and technical features in precision crop breeding.

Editing Technology	Main Effector or Editing Module	Cas/Scaffold Protein	Main Guide or Targeting Element	Editing Output	Main Advantages	Key Limitations	**References**
ZFNs and TALENs	Engineered DNA-binding proteins fused with FokI nuclease domains	FokI nuclease dimer	Protein-based DNA recognition modules	Targeted DSBs followed by NHEJ or HDR	Early programmable nuclease platforms for plant genome modification	Target redesign requires protein engineering, limiting scalability and multiplexing	[42,43,44]
CRISPR–Cas nuclease editing	RNA-guided DNA endonuclease	Cas9, Cas12a or engineered variants	sgRNA or crRNA plus PAM	Indels and gene knockout; donor-mediated insertion or replacement through HDR	Simple, efficient and multiplexable	PAM dependence, possible off-target cleavage, DSB-associated by-products and low HDR efficiency in plants	[9,42,43,45]
TnpB-mediated genome editing	TnpB RNA-guided endonuclease	TnpB	ωRNA plus TAM	Targeted DNA cleavage and indel mutagenesis	Ultra-compact and compatible with viral or RNP delivery	Orthologue-, TAM- and target-dependent activity; validation remains limited across crops	[56,57,58,59,60,61]
Canonical base editing (CBE and ABE)	Cytidine or adenine deaminase	nCas9(D10A) or engineered Cas variants	sgRNA or crRNA plus PAM	C:G-to-T:A or A:T-to-G:C substitutions	Precise transition editing without DSBs or donor DNA	PAM and editing-window constraints, bystander edits and possible off-target deamination	[4,6,14,42,62]
Prime editing	Reverse transcriptase editing module	nCas9(H840A)–reverse transcriptase fusion	pegRNA containing spacer, PBS and RTT; optional nicking sgRNA	All 12 base substitutions and precise small insertions or deletions	Versatile sequence rewriting without DSBs or a separate donor template	Variable and locus-dependent efficiency, complex pegRNA design and large editor size	[5,6,15,64,65]
Large-fragment genome engineering	Paired Cas nucleases, dual-prime-editing, or recombinase/transposase-assisted modules	Cas nuclease(s), prime editor and/or site-specific recombinase or transposase	Paired sgRNAs or pegRNAs; donor DNA and auxiliary recognition sites when required	Large deletions, insertions, replacements, inversions, duplications or translocations	Enables structural-variant engineering, trait stacking and linkage redesign	Low or variable efficiency, difficult large-cargo delivery, unintended rearrangements or junction errors and complex validation	[8,43,68]
CRISPR activation and interference (CRISPRa/i)	Transcriptional activators, repressors or dCas-mediated steric blockade	dCas9, dCas12a or an effector-recruitment scaffold	sgRNA or crRNA targeting promoters or regions near the TSS	Activation or repression of endogenous gene transcription	Tunable and multiplexable expression control without changing DNA sequence	Strong dependence on guide position, promoter architecture, chromatin state and editor expression	[72,73,74,75,76,77]
Epigenome editing	DNA methylation, demethylation or histone-modification effectors	dCas9–effector fusion or multicomponent recruitment scaffold	sgRNA targeting regulatory or chromatin-associated loci	Locus-specific DNA methylation, demethylation or histone-mark changes	Sequence-preserving gene regulation and potential epiallele generation	Efficiency, specificity, persistence and inheritance are locus-, mark- and editor-dependent; epigenetic off-target effects require evaluation	[9,10,71,78]
RNA targeting and editing	Cas13 RNase or dCas13-linked RNA-modifying effector	Cas13 or dCas13–effector fusion	crRNA targeting the transcript	Transcript cleavage or knockdown; RNA base editing when coupled to an RNA modifier	Reversible transcript regulation without altering genomic DNA	Delivery and expression dependence, transient activity, variable efficiency and potential collateral RNA cleavage	[9,80,81]
Organelle base editing	Cytidine deaminase, FokI nickase and exonuclease	Organelle-targeted TALE-based CyDENT editor	Paired TALE DNA-binding arrays	Strand-preferred C:G-to-T:A editing in mitochondrial or chloroplast DNA	Guide-RNA-free access	Sequence-context and strand constraints, delivery and regeneration challenges, and difficulty obtaining stable homoplasmic lines	[7]

## 3. Delivery Systems and Generation of Transgene-Free Edited Germplasm

Plant genome-editing efficiency is governed by more than editor activity alone [2,3,82]. Delivery of editing reagents, recipient tissue status, tissue culture conditions and plant regeneration capacity jointly affect whether edited events can be obtained and stably inherited [82]. Current delivery approaches for crop genome editing can be broadly grouped into DNA-based transformation and transient or transgene-free delivery systems [82]. *Agrobacterium*-mediated transformation and particle bombardment remain the main DNA-based routes for stable expression of editing reagents, although bombardment can cause multicopy insertion and genome-scale sequence disruption [82,83]. RNP and transient DNA/RNA delivery are used to reduce foreign-DNA integration and shorten editor exposure [84,85,86]. Viral vectors, grafting-based delivery and tissue-culture-free strategies provide complementary routes for heritable or transgene-free editing in selected crops [87,88,89,90,91]. Protoplast and hairy-root systems are useful for gRNA testing and rapid functional validation, but editing efficiency in these systems does not necessarily reflect the efficiency with which independent and fertile edited plants can be recovered, particularly in genotypes with limited regeneration capacity [55,82].

RNP or RNA delivery avoids the use of DNA-encoded editing constructs, whereas transient DNA delivery can also yield transgene-free edited plants when the editing cassette is not stably integrated; when stable integration occurs, transgene-free progeny may still be recovered through segregation [84,85,86]. The recovery of transgene-free edited germplasm is an important objective for translating genome editing into crop breeding [82]. RNP delivery introduces preassembled Cas protein–gRNA complexes directly into plant cells, thereby avoiding DNA-encoded editing reagents and shortening intracellular editor exposure [84]. This approach reduces the likelihood of foreign DNA integration and may decrease certain off-target effects [84,85,86]. Preassembled CRISPR–Cas9 RNPs have been used for DNA-free editing in *Arabidopsis*, tobacco, lettuce and rice, with regenerated edited plants demonstrating the feasibility of recovering DNA-free germplasm [84,86]. Subsequent studies extended RNP-mediated editing to maize and bread wheat, including delivery through particle bombardment, supporting the feasibility of this strategy in major crops [85,86]. Transient expression of CRISPR/Cas9 DNA or RNA in wheat has also produced transgene-free edited materials, providing a complementary route for editing strategies that do not rely on stable integration [86]. However, RNP or transient delivery generally requires protoplast regeneration, embryogenic tissue culture or efficient bombardment platforms, and its performance remains highly dependent on species- and genotype-specific regeneration capacity [82,84,85,86]. Thus, the advantage of DNA-free or transient delivery ultimately depends on whether edited cells can be regenerated into fertile and heritable plants in the target genotype [82,84,85,86].

Low transformation efficiency and limited regeneration capacity remain major technical barriers to the direct application of genome editing in elite commercial cultivars [82]. In wheat, soybean and cotton, readily transformable genotypes often differ substantially from breeding target genotypes [82]. For practical breeding, this genotype dependence is particularly important because editing an easily transformable donor genotype does not directly produce an edited version of the desired elite cultivar. Improving transformation and regeneration in breeding-target genotypes can therefore shorten the route from allele creation to usable edited germplasm and reduce reliance on subsequent introgression [82]. Recent optimization of crop editing systems has consequently shifted from reagent delivery alone toward coordinated control of delivery, cell fate reprogramming and regeneration [82]. Viral vectors provide an alternative route to reduce tissue culture dependence [87,88,89,91]. RNA virus-mediated delivery has enabled editing in systems such as tobacco, pepper and wheat [88,89,92,93]. Grafting wild-type shoots onto transgenic donor rootstocks expressing mobile CRISPR reagents has also generated transgene-free edited progeny without direct transformation of the target shoot, offering a potential strategy for perennial or transformation-recalcitrant crops [90]. These approaches expand the range of possible delivery strategies for difficult-to-transform germplasm, although their applicability depends on factors such as host range, movement of editing reagents into meristematic or reproductive tissues, and transmission of edited alleles to the next generation [87,88,89,90,91,92,93].

Developmental regulators provide an important strategy for overcoming regeneration bottlenecks [94,95,96]. *Baby boom* (*BBM*) and *Wuschel* (*WUS*) promote cell division, somatic embryogenesis and meristem maintenance, thereby improving transformation efficiency in recalcitrant monocot crops such as maize and showing cross-species utility in sorghum, sugarcane and rice [94]. The *GRF–GIF* chimeric protein enhances regeneration speed and broadens the range of transformable genotypes in wheat, rice, citrus and other plants by modulating growth- and development-related transcriptional networks [95]. In the reported systems, *GRF–GIF* supported efficient regeneration with limited obvious developmental abnormalities or fertility defects [95]. *TaWOX5* has also been shown to enhance transformation and regeneration capacity in wheat, triticale, rye, barley and maize, further supporting the value of *WUS*/*WOX*-related factors in reducing genotype dependence [96]. These regeneration-promoting factors alleviate genotype dependence mainly by increasing the developmental competence of transformed or edited cells. *BBM/WUS*- and *WOX*-related regulators promote embryogenic or meristematic development, whereas *GRF-GIF* enhances cell proliferation and regenerative competence. As a result, cells from genotypes that respond poorly to conventional tissue-culture systems have a greater probability of regenerating into whole plants [94,95,96]. Their breeding value therefore lies not only in increasing regeneration efficiency, but also in broadening the range of genotypes in which edited plants can be recovered, including breeding-target and potentially elite germplasm [82,94,95,96].

The isopentenyl transferase gene *ipt* represents a distinct regeneration-promoting module associated with cytokinin biosynthesis and has recently attracted attention in in planta meristem induction and tissue culture-independent editing systems [97,98,99,100]. In contrast to *BBM*, *WUS* and *GRF–GIF*, which mainly act as transcriptional regulators, *ipt* increases local cytokinin levels and promotes cell proliferation, shoot primordium formation and meristem reconstruction [97,98,99,100]. These properties allow *ipt* to be combined with developmental regulators or wound-induced signaling modules to promote de novo meristem formation and recovery of edited shoots [97,98,99,100]. Co-delivery of *WUS2* and *Agrobacterium ipt* can induce de novo meristems in somatic tissues and generate heritable edited progeny [97]. In tomato, RNA viral delivery of *ipt* together with sgRNAs promoted the formation of edited shoots associated with latent axillary meristematic cells, enabling heritable editing with reduced tissue culture dependence [98]. A *WIND1–ESR1–ipt* synthetic transcriptional cascade further demonstrated that *ipt* can function as a cytokinin-driven organogenesis module coupled with wound-induced reprogramming for in planta regeneration and genome editing in tobacco, tomato and soybean [99]. In Chinese cabbage, combined expression of *WUS2*, *IPT* and *PLT5* also indicated that coordinated expression of multiple regeneration-promoting factors may improve transformation and editing efficiency in recalcitrant horticultural crops [100]. Together, these studies show that local hormonal regulation can complement morphogenic regulators by increasing the probability that edited cells establish regenerating shoots or meristems.

Ectopic expression of developmental regulators and cytokinin biosynthesis factors can cause abnormal differentiation, chimerism, dwarfism, excessive branching, leaf deformation or reduced fertility [94,95,97,99,100]. Their application should therefore be viewed as engineered control of recipient cell fate, hormonal homeostasis and meristem establishment rather than as a simple enhancement of regeneration efficiency [94,95,97,99]. By broadening the range of genotypes capable of regeneration, these approaches can also facilitate the direct recovery of edited plants from breeding-target or elite germplasm and reduce dependence on highly regenerable donor backgrounds [82,94,95,96]. Future strategies with greater translational potential should restrict the spatiotemporal activity of regeneration-promoting factors through inducible promoters, tissue-specific expression, transient expression, removable vectors or viral transient delivery [82,89,98,99]. Integration of these approaches with RNP or RNA delivery, efficient genotyping and transgene-free progeny screening will be essential for improving the recovery of heritable edited events while reducing developmental perturbation and foreign DNA retention [82,84,86,89,90]. For breeding applications, the important endpoint is the recovery of independent, fertile, non-chimeric and heritable edited plants in the target genetic background, rather than regeneration frequency alone. Overall, the development of transgene-free edited crop germplasm is shifting from the optimization of individual delivery methods toward the coordinated integration of delivery vehicles, recipient tissues, regeneration control and progeny screening [82,94,95,99,100]. Progress in this area will depend on improving the recovery of heritable edited events, minimizing foreign DNA integration and developmental abnormalities, and establishing robust editing pipelines applicable to diverse crops, genotypes and elite commercial cultivar backgrounds, thereby facilitating the deployment of genome-edited materials in practical breeding [82,94,95,99].

Unintended genetic outcomes are not limited to conventional off-target substitutions or indels. DSB-based editing can generate large deletions or chromosomal rear-rangements at the intended locus, DNA delivery can result in vector or DNA-fragment integration, and prolonged tissue culture can introduce somaclonal variation [3,8,42,43,82,83]. Event characterization should therefore consider both sequence-level and structural changes, and independent edited events or segregated progeny are important for separating programmed edits from transformation- or culture-associated variation [3,82,83].

## 4. Applications of Genome Editing in Crop Trait Improvement

Breeding relevance depends on the level of phenotypic evidence. Molecular or greenhouse phenotypes can establish gene function or proof of concept, whereas agronomic benefit requires stable inheritance and replicated evaluation under field conditions [2,3,101,102]. ARGOS8 regulatory alleles in maize and ultra-low-asparagine wheat provide field-tested examples, while many reported edits remain at controlled-environment or early-generation stages [101,102]. Quantitative traits require multi-environment testing because genetic background, management and genotype-by-environment interactions can alter effect size and stability [29,35,103].

### 4.1. Genome Editing for Ideotype Design and Yield Improvement

**Figure 2:**
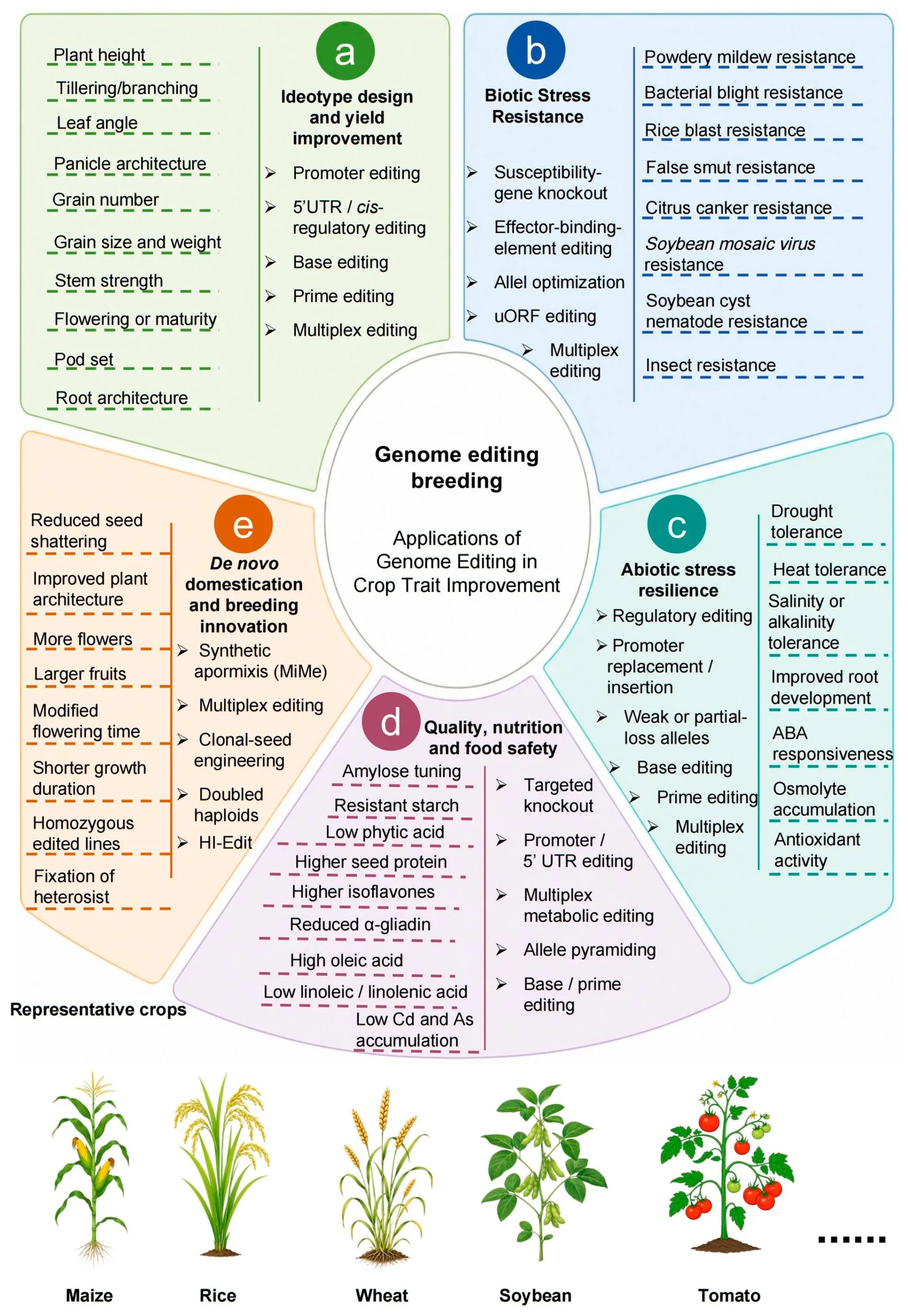
Applications of genome editing in crop trait improvement. (**a**) Ideotype design and yield improvement. (**b**) Biotic stress resistance. (**c**) Abiotic stress resilience. (**d**) Quality, nutrition, and food safety. (**e**) Domestication and breeding innovation. Ellipses denote additional crop species not shown.

Plant architecture is a major determinant of crop performance because it governs resource capture, biomass allocation and canopy function [1]. Traits such as plant height, tillering or branching capacity, leaf angle, inflorescence morphology, grain or seed size, stem strength and root architecture collectively influence light interception, canopy aeration, lodging resistance, planting density, mechanized management and harvest index [1] (Figure 2a). The deployment of semi-dwarf alleles, represented by *sd1* in rice and *Rht* in wheat, was central to the Green Revolution and showed that modifying plant stature can substantially enhance yield potential [1,17,18,19]. Modern ideotype breeding, however, goes beyond height reduction and aims to coordinate developmental and physiological processes that shape canopy structure, assimilate production and adaptation to cultivation systems [1,17]. Genome editing offers a precise way to modify key regulatory loci and reconfigure architectural and physiological trait modules [2,17,18,19,20]. Because yield emerges from interactions among source capacity, sink strength, assimilate transport and field adaptation, edited alleles should be evaluated in relation to source–sink coordination and target production systems [1,17]. Promoter, 5′UTR and other *cis*-regulatory editing strategies provide practical routes for generating tunable alleles rather than isolated null mutations [16,18,19,20].

In rice, well-characterized yield-related loci such as *Gn1a/OsCKX2*, *DEP1*, *GS3*, *GW2* and *IPA1/OsSPL14* have become important targets for genome-editing-based improvement of grain number, panicle architecture, grain size, grain weight, tillering and ideal plant architecture [17,19,20]. Editing negative regulators can enhance individual yield components, but null mutations are not always agronomically optimal because complete loss of function may disrupt developmental coordination. *IPA1/OsSPL14* illustrates this complexity: beneficial alleles can increase panicle size and strengthen culms, whereas excessive *IPA1* activity may suppress tillering in certain backgrounds [17]. Promoter editing generated the *IPA1-Pro10* allele, which reshaped spatiotemporal expression rather than abolishing gene function, increasing grain number while maintaining suitable tillering [17]. *Cis*-regulatory editing can provide finer control of complex yield traits than complete coding-sequence disruption [17,19,20].

For many yield-related loci, desirable breeding alleles require calibrated changes in gene expression or protein activity rather than complete loss of function [16,17,19,20]. Editing non-coding regulatory regions, including promoters, 5′ untranslated regions, upstream open reading frames and 3′ untranslated regions, can generate allelic series with graded phenotypic effects by modulating transcription, translation or mRNA stability [16,19,21,22]. In parallel, base editing and prime editing enable precise amino acid substitutions that adjust protein activity with limited disruption of gene function [5,6,14,15]. In maize, promoter editing of *CLE* genes produced regulatory alleles with distinct effect sizes and improved kernel row number and other yield-related traits [18]. In rice, 5′UTR editing generated graded reductions in gene expression and continuous variation in plant height, tiller number and leaf angle [19]. Yield-oriented genome editing is consequently shifting from null-allele generation toward tunable quantitative alleles [16,18,19,22].

In soybean, yield potential is shaped by an integrated network of architectural, phenological and physiological traits, including plant stature, flowering and maturity, internode elongation, leaf development and source–sink balance [49,51,52,54,55]. Loci such as *GmFT2a*, *GmFT5a*, *E1*, *GmEID1*, *GmPRR37*, *GmPRR3b* and *GmLNK2* regulate flowering time, maturity and latitudinal adaptation, while genes involved in vegetative architecture and assimilate production are also emerging as editing targets [49,51,52]. For example, disruption of *GmLHY* family members alters gibberellin metabolism and reduces plant height and internode length, editing of *GmPPD1*/*GmPPD2* modifies leaf and seed size, and *GmSPL9* family members have been associated with plant architecture and yield-related traits [53,55]. Because soybean has a paleopolyploid origin and retains many duplicated genes and homologous gene families, single-gene mutations may be buffered by functional redundancy and show limited breeding value [54,55]. Multiplex editing is therefore particularly important for uncovering masked gene functions and coordinating architectural and physiological trait modules to improve yield potential [54,55].

Because grain yield depends on the coordinated action of canopy architecture, reproductive sink formation and assimilate supply, genome-editing strategies should move beyond the optimization of individual traits [17,18,19]. Dense planting systems generally favor compact plant type, stronger stems and improved lodging resistance, but excessive reduction in plant stature may limit radiation capture, biomass accumulation and yield potential [1,17]. Expanding sink capacity through increased grain number, pod set or branching can improve productivity only when source capacity and assimilate transport are sufficient to support seed set and grain filling [17]. Otherwise, larger sink demand may be offset by reduced fertility, incomplete grain filling or lower seed weight [17]. Edited alleles should therefore be designed in relation to the target production environment, including planting density, mechanization requirements, ecological adaptation and source–sink coordination [1,17]. Integrating multiplex genome editing with natural-variation mining, high-throughput phenotyping and artificial-intelligence-assisted prediction may help identify allele combinations with stable field performance and greater breeding utility [29,30].

### 4.2. Genome Editing for Durable Resistance to Biotic Stress

Biotic stress from pathogens and insect pests remains a major constraint on crop productivity, yield stability and product quality [104,105]. Conventional resistance breeding relies on resistant germplasm, introgression and pyramiding of resistance loci, but its durability can be weakened by pathogen race shifts, pest adaptation, linkage drag and genetic-background effects [105,106,107,108]. Genome editing complements this approach by enabling targeted modification of host factors involved in plant–pathogen and plant–insect interactions [2,3,23,105]. Modification of susceptibility factors limits host functions exploited by pathogens, whereas editing of resistance genes or immune receptors can alter pathogen recognition, and modulation of immune-related regulators can adjust the strength or duration of defense responses [23,25,107,109]. Regulatory editing offers opportunities to adjust target-gene expression rather than eliminate gene function, while multiplex editing enables combinations of changes at multiple loci for resistance improvement [23,25,107,109]. The resulting resistance spectrum and agronomic effects depend on both the functions of the targeted genes and the alleles generated, so editing strategy can influence the balance between enhanced defense and penalties to growth, development and yield [25,104,105,109] (Figure 2b).

Editing susceptibility genes provides a direct route to disease resistance by limiting host functions exploited by pathogens [25,106,107,109]. The wheat *MLO* locus is a classic example, as loss-of-function mutations in *MLO* homoeologs confer broad-spectrum powdery mildew resistance [106,107]. However, complete *MLO* disruption may cause premature senescence, reduced plant height or yield penalties [107]. The Tamlo-R32 line represents a more refined resistance allele, retaining powdery mildew resistance without detectable growth or yield defects [25,107,109]. Mechanistic studies showed that an approximately 304 kb deletion near *TaMLO-B1* alters local three-dimensional chromatin organization and activates the upstream gene *TaTMT3*, which may buffer the negative effects of *MLO* loss of function [107]. Resistance allele design should therefore consider both resistance strength and the developmental consequences of the edited genomic context [25,107,109].

*Cis*-regulatory editing offers another way to reduce host susceptibility while preserving basal gene function [20,23,108]. In rice, *Xanthomonas oryzae pv. oryzae* activates *SWEET* sugar transporter genes by using transcription activator-like effectors to bind effector-binding elements in their promoters, thereby promoting bacterial colonization [23]. Editing these promoter elements uncouples *SWEET* expression from pathogen effector activity and confers broad-spectrum resistance to bacterial blight [23,25]. A similar strategy has been applied in citrus by modifying effector-binding sites in the *CsLOB1* promoter to enhance resistance to citrus canker [104,108]. By blocking pathogen-induced transcriptional activation while retaining endogenous gene function, *cis*-regulatory editing can improve resistance with fewer pleiotropic effects than coding-sequence knockout [20,23,108].

Because immune regulation is tightly linked with plant growth, development and metabolism, complete disruption of resistance-related genes may compromise agronomic performance [25,104,105,109]. For pleiotropic or dosage-sensitive loci, desirable alleles are more likely to result from calibrated modulation than from full loss of function [20,25,109]. Saturation mutagenesis and allele mining are therefore useful for identifying variants that strengthen defense while maintaining developmental balance [20,25,109]. In rice, strong impairment of *RBL1*, which encodes a cytidine diphosphate-diacylglycerol (CDP-DAG) synthase, disrupts growth, whereas the engineered *RBL1Δ12* allele improves resistance to rice blast, false smut and bacterial blight without an obvious yield penalty in small-scale field trials [25,109]. Editing upstream open reading frames provides another means of tuning defense output by altering translational efficiency while preserving basal gene function [109]. These studies support an allele-optimization framework in which genome editing fine-tunes resistance-related genes rather than defaulting to null mutations [20,25,109]. In soybean, genome editing has mainly been used to link candidate genes with resistance phenotypes and generate experimental germplasm [55]. Multiplex mutagenesis of *GmF3H1*, *GmF3H2* and *GmFNSII-1* redirects flavonoid metabolism toward higher isoflavone accumulation and improves resistance to *Soybean mosaic virus* (SMV) [110]. For soybean cyst nematode, CRISPR-based perturbation of resistance-associated targets, often combined with hairy-root transformation, has enabled rapid evaluation of candidate loci and helped dissect host–nematode interactions [55,104,105].

Genome editing for insect resistance is more complex than many host–pathogen systems because pest damage is influenced by feeding mode, host-location cues, detoxification capacity, defensive chemistry, epidermal and cell-wall traits, volatile-mediated interactions and field ecology [2,3,104,105]. Edited resistance is therefore unlikely to rely on a single universal target [2,3,105]. More realistic strategies include attenuating host factors that facilitate feeding or colonization, redirecting defensive secondary metabolism, reinforcing structural barriers, or combining edited traits with Bt protection, RNA interference, induced resistance and biological control [104,105]. Controlled inoculation, artificial infestation and detached-tissue assays can establish mechanistic relevance, but breeding value ultimately requires field evidence for resistance durability, yield stability, compatibility with integrated pest management and limited effects on non-target organisms across environments with different pest pressures [104,105].

### 4.3. Genome Editing for Abiotic Stress Resilience

Environmental constraints, including drought, salinity–alkalinity, temperature extremes and nutrient limitation, strongly influence crop adaptation and yield stability [101,103,111]. Unlike some host–pathogen interactions governed by major resistance loci, abiotic-stress resilience is usually quantitative and environment-dependent, involving coordinated regulation of root development, stomatal behavior, hormonal and redox signaling, ion transport, phenology and source–sink relations [111]. Genome editing should therefore be used not simply to knock out putative stress-resistance genes, but to remodel regulatory nodes that constrain crop performance in defined target environments [2,101,103,112]. Expression tuning, weak or partial-loss-of-function alleles and multiplex editing provide practical routes to adjust stress-response pathways while balancing tolerance, growth and productivity [2,103,112,113] (Figure 2c). A single edited locus should therefore be interpreted as evidence for a specific stress-related trait rather than as evidence of stable field tolerance across environments, because its phenotypic effect can vary with stress intensity, developmental stage and genetic background [103,111].

Field studies in maize show that targeted regulatory editing can improve yield under defined drought conditions [101,103]. CRISPR–Cas-mediated replacement or insertion of the *GOS2* promoter at the *ARGOS8* locus increased *ARGOS8* expression and improved grain yield under flowering-stage water stress, without yield penalties under well-watered conditions [101,103]. This supports regulatory allele engineering as a strategy to improve crop performance under defined water-stress conditions while maintaining productivity under well-watered conditions [101,103]. Temperature tolerance involves distinct regulatory and physiological mechanisms. In rice, the NAT1–bHLH110–CER1/CER1L module and a natural gene on–off system have been associated with heat-stress responses and field thermotolerance, respectively [112,113]. However, stress-adaptive traits often involve trade-offs: constitutive stress activation may restrict growth, root architectural changes can alter water and nutrient acquisition, and flowering-time shifts may affect regional adaptation [103,111]. Abiotic-stress improvement is therefore better suited to graded or conditional modulation of gene activity through promoter editing, base editing, prime editing or tissue- and environment-responsive regulation [2,103,112,113]. The breeding value of such alleles should be validated through multi-year and multi-location field trials in relevant target environments, rather than inferred only from controlled stress assays [103,112,113,114].

In soybean, CRISPR/Cas editing has begun to identify regulators associated with drought, salinity–alkalinity and heat responses, although much of the current evidence comes from hairy-root, chimeric or early-generation materials [55,114]. CRISPR perturbation of *GmMYB118* in hairy roots reduced drought and salt tolerance, whereas overexpression improved both responses, supporting its positive role in osmotic and salt-stress adaptation [115]. Simultaneous disruption of *GmSnRK1.1* and *GmSnRK1.2* altered ABA responsiveness and weakened resistance to NaHCO_3_-induced alkaline stress, implicating these *SnRK1* homologues in hormone-linked alkaline-stress regulation [116]. Editing *GmHdz4* in hairy-root chimeras enhanced root development, osmolyte accumulation and antioxidant activity under PEG-simulated drought stress, whereas *GmHsp90A2* knockout reduced thermotolerance [117,118]. These genes provide useful candidates for soybean stress-resilience breeding, but their agronomic value still needs confirmation in elite backgrounds and multi-environment field trials [55,103,114].

### 4.4. Genome Editing for Crop Quality, Nutritional Value, and Food Safety

Crop improvement is now judged not only by yield potential but also by the quality, nutritional value and safety of harvested products [2,3]. Rice grain-quality editing and CoQ10 engineering illustrate improvement of starch and nutritional traits through targeted editing [21,22,24]. Wheat examples show that genome editing can alter gluten or asparagine-related food-safety traits [50,102]. Soybean studies have targeted fatty acid composition, phytic acid, seed protein and isoflavone-related quality traits [119,120,121,122]. Food-safety applications include regulatory editing of *OsNRAMP5* and pyramiding of *OsNramp5*/*OsLsi2* alleles to reduce heavy-metal accumulation [123,124]. Compared with repeated donor introgression, targeted editing can reduce linkage drag, shorten quality improvement cycles and better preserve agronomic performance [2,3] (Figure 2d). Quality-oriented genome editing increasingly targets genes involved in interconnected metabolic and transport pathways, in which modification of a single component can influence not only the desired product composition but also broader physiological and agronomic responses [2,3,22,123]. The magnitude of these effects depends on the function of the target gene and the type of allele generated [16,19,20,21,22,123]. Strong loss-of-function mutations may produce pronounced compositional changes, whereas regulatory or partial-function alleles can provide more graded control of pathway activity and may reduce undesirable effects on associated traits [19,20,21,22,123]. Such allele-level optimization is therefore particularly relevant when breeding objectives require simultaneous improvement of product quality and maintenance of agronomic performance [2,3,22,123,125].

Rice grain quality illustrates the value of quantitative allele design [21,22,24,125]. Loss-of-function mutations in starch-biosynthetic genes such as *Wx* and *SBEIIb* can substantially alter amylose content, amylopectin structure or resistant starch accumulation, but strong disruptions may compromise grain filling, yield or palatability [21,22]. In contrast, promoter or 5′UTR editing of *Wx* has generated graded amylose variation and improved grain quality, while moderate editing of *SBEIIb cis*-regulatory elements increased resistant starch content with limited effects on major agronomic traits [21,22]. In wheat, multiplex editing of α-gliadin genes reduced the immunoreactive gluten fraction, showing that genome editing can also reshape processing and health-related quality traits [102,119,125]. Field trials of ultra-low-asparagine genome-edited wheat further extended quality and food-safety evaluation to field performance and baking characteristics [102].

Oil quality is among the most established genome-editing targets in soybean [50,120]. Editing fatty acid desaturase genes, including *GmFAD2-1A*/*GmFAD2-1B* or *GmFAD2-2*, increases oleic acid content while reducing linoleic and linolenic acids, thereby improving oxidative stability and processing quality of soybean oil [50,120]. Other quality-related targets include *GmIPK1* for lowering phytic acid, seed-protein or *NF-YC4* regulatory modules for modifying seed protein composition, and *GmF3H1*, *GmF3H2* and *GmFNSII-1* for redirecting flavonoid metabolism toward higher isoflavone accumulation [110,121,122]. Because soybean quality traits often involve duplicated genes and interconnected metabolic branches, effective improvement usually requires coordinated editing rather than modification of a single locus [50,110,120,121,122].

Food-safety traits are also well suited to precise allele design, as small changes in transporter activity or expression can markedly alter the accumulation of undesirable compounds [102,123,124]. In rice, *OsNRAMP5* knockout reduces grain cadmium accumulation but may disturb manganese uptake and plant growth, whereas regulatory-region editing of *OsNRAMP5* lowers cadmium accumulation while better maintaining manganese homeostasis and agronomic performance [102,123,124]. Pyramiding edited *OsNramp5* and *OsLsi2* alleles further produced two-line hybrid rice with ultra-low cadmium and arsenic accumulation under contaminated field conditions [124]. Therefore, food-safety editing should be evaluated not only by reductions in toxic elements, but also by mineral nutrient balance, yield performance, environmental adaptation and processing quality [102,123,124].

### 4.5. Genome Editing–Enabled De Novo Domestication and Breeding System Innovation

Beyond improving individual traits in established cultivars, genome editing is broadening the ways in which breeding materials are generated, diversified and fixed [2,3]. Although domestication and selection have increased crop productivity and adaptation, they have also narrowed genetic diversity and separated elite cultivars from useful alleles for stress tolerance, disease resistance and environmental adaptation [126,127,128,129]. De novo domestication offers a complementary strategy by directly editing domestication- or improvement-related genes in wild relatives, orphan crops or underutilized species, thereby improving agronomic performance while retaining valuable wild variation [126,127,128,129]. In wild tomato and *Solanum pimpinellifolium*, multiplex editing of genes involved in plant morphology, flowering, fruit traits and nutritional quality generated domesticated phenotypes while preserving useful stress-resistance, disease-resistance, or quality-related traits [125,126,127]. In groundcherry, editing orthologues of tomato domestication genes improved plant architecture, flower production and fruit size, demonstrating the transferability of knowledge from model crops to orphan crop improvement [128]. In wild allotetraploid rice, efficient transformation and editing systems enabled rapid modification of seed shattering, plant height and growth duration, supporting genome editing as a route for polyploid wild crop domestication [129]. Given the reliance of global food systems on a limited number of staple crops, orphan and underutilized species remain important reservoirs of nutritional diversity, adaptive traits and genetic variation [2,126,127,128,129]. Because domestication syndromes involve multiple traits, including seed shattering, flowering time, plant architecture and fruit or seed size, multiplex editing can accelerate the coordinated modification of several domestication-related loci, as demonstrated in wild tomato and allotetraploid rice [126,127,129]. However, pleiotropic effects, epistatic interactions and genetic-background dependence can complicate the phenotypic consequences of combined edits, making it necessary to evaluate edited allele combinations together with pre-existing adaptive and agronomic traits [127,130]. Pangenome resources can complement this process by revealing accession-specific genes, structural variants and haplotypes that may be absent from a single reference genome, thereby improving target and guide selection across genetically diverse wild germplasm [29,31,32,130]. The retention of parental disease resistance and salt tolerance in edited wild-tomato accessions further illustrates the importance of preserving favorable wild-background variation while modifying domestication-related loci [127]. Together, studies in wild tomato, groundcherry and allotetraploid rice indicate that de novo domestication is better viewed as the selective redesign of key domestication traits within genetically valuable wild backgrounds rather than as indiscriminate multi-gene knockout [126,127,128,129].

Genome editing is also being integrated with haploid induction and doubled haploid technologies to accelerate fixation of favorable genotypes [26,130,131,132,133]. Doubled haploid breeding shortens the production of homozygous lines, while haploid-induction pathways involving *MTL*/*PLA1*/*NLD*, *DMP* and *CENH3* provide key entry points for engineering haploid-induction systems [131,132,133]. HI-Edit combines haploid induction with CRISPR/Cas editing, allowing editing reagents to act on the target parental genome during haploid induction and enabling edited haploids to be recovered in a single workflow [26]. In maize, HI-Edit produced edited haploid plants lacking both haploid-inducer parental DNA and editing machinery, reducing the need for direct transformation of elite germplasm [26]. Editing followed by doubled haploid production further combines multiplex editing, in vivo haploid induction and chromosome doubling to generate homozygous edited maize lines within a shortened breeding cycle [26,130,131,132,133]. Wider application of these systems is still constrained by haploid-induction efficiency, editor delivery, genotype responsiveness and reliable chromosome doubling [26,130,131,132,133].

Synthetic apomixis provides another route for preserving elite heterozygous genotypes and fixing hybrid vigor through seed [27,130,134]. The MiMe system redirects meiotic inheritance toward mitosis-like gamete formation by modifying meiosis-related genes, producing unreduced clonal gametes [27,130,134]. When combined with parthenogenesis- or haploid-induction-related components, clonal seed production from hybrid genotypes becomes experimentally feasible [27]. In rice F1 hybrids, simultaneous editing of *REC8*, *PAIR1*, *OSD1* and *MTL* produced clonal seeds, demonstrating that genome editing can reprogram meiosis- and fertilization-related processes [27]. In tomato, MiMe generated unreduced clonal gametes, and fusion of these gametes enabled the construction of polyploid materials carrying four predefined haplotypes [134]. These studies highlight the potential of synthetic apomixis and clonal-gamete-based strategies for heterozygous genotype fixation and polyploid genome design [27,130,134].

Together, these advances extend crop genome editing from single-allele creation to germplasm construction, genotype fixation and breeding-system redesign [26,27,130,134] (Figure 2e). Their practical value will ultimately depend on whether edited alleles and allele combinations perform consistently in target cultivar backgrounds and real production environments [2,3].

### 4.6. Genome Editing in Polyploid Crops

Polyploid crop editing is influenced by homoeolog redundancy and allele dosage, so the phenotype depends on both the identity and number of edited copies [2,3,54,55,106]. Simultaneous editing of multiple *MLO* homoeoalleles in hexaploid wheat was required for strong powdery mildew resistance [106]. Dose–response relationships can nevertheless differ among loci. CRISPR-Cas9 editing of *TaGW2* homoeologues produced largely additive effects on grain size and thousand grain weight, with different homoeologues contributing unequally in different cultivars [135]. In contrast, *TaGS3* editing showed non-additive dosage effects: complete knockout of all three homoeologous copies reduced grain size and weight, whereas selected single- or double-genome mutant combinations increased these traits [136]. These contrasting outcomes illustrate why editing more copies does not necessarily produce a larger or more favorable phenotype in polyploid crops.

Guide design in polyploids therefore involves two distinct objectives. Pan-homoeolog guides exploit conserved sequences to mutate several copies simultaneously, whereas homoeolog-specific guides depend on diagnostic SNPs, indels or PAM differences to modify selected copies and create dosage series. Copy-resolved reference assemblies and pangenomes can improve both strategies by identifying conserved target sites, copy-specific polymorphisms and potential off-target sequences [29,31,32]. Multiplex editing can overcome functional redundancy efficiently, but it also increases the number of edited alleles that must be genotyped, fixed and phenotypically interpreted across generations. For dosage-sensitive or pleiotropic loci, partial or homoeolog-specific editing may therefore provide more predictable breeding outcomes than complete multi-copy knockout [29,31,54,55,135,136].

## 5. Integration of Artificial Intelligence with Crop Genome Editing

Artificial intelligence is making crop genome-editing design more predictive by shifting part of the workflow from empirical screening to data-assisted prioritization [30]. Machine-learning and deep-learning models can support target discovery, guide design, editor selection and allele-performance prediction by integrating sequence context, chromatin features and editing outcomes [30,36,37]. Pangenome, multi-omics, phenotypic and environmental datasets can further refine candidate prioritization for complex traits [29,30,35]. Protein-structure prediction and generative protein design may contribute to future editor engineering, although plant-cell validation remains essential [38,39,40,41]. In precision breeding, these approaches can reduce the experimental search space before validation rather than replace biological testing [30,36,37]. Model performance remains limited by the scarcity of plant-specific training data, uneven transferability across species and genotypes, incomplete representation of chromatin and epigenetic context, and limited incorporation of environmental effects [30,36,37].

At the guide RNA design stage, AI models use PAM compatibility, guide-sequence features, GC content, chromatin accessibility and genome-wide similarity to predict on-target activity and off-target risk [30,36,37]. In polyploid crops, guide selection also requires homoeologue-resolved annotation and pangenome resources to distinguish guides for simultaneous editing of all copies from those for copy-specific editing [9,29,30]. For base editing, target-library-derived models can predict editing efficiency, product distribution, bystander edits and indel by-products across editor architectures [4,36,42]. For prime editing, deep-learning models have been developed to predict pegRNA performance, intended-edit efficiency, product purity and chromatin-associated variation in editing outcomes [37,64,65]. These models are useful because editing outcomes are shaped jointly by editor architecture, local sequence context, guide or pegRNA design, DNA repair activity and chromatin state, rather than by the target sequence alone [30,36,37]. However, many widely used prediction models were developed primarily from non-plant datasets, and their transferability to crop species cannot be assumed. Plant-specific benchmarking is beginning to address this limitation. In *Nicotiana benthamiana*, 52 experimentally tested gRNAs were used to evaluate more than 20 computational tools for SpCas9 activity prediction; several machine-learning-based models showed significant correlations with in planta editing efficiency, but predictive performance varied between experimental datasets [137]. The limited number of gRNAs and the use of a single plant species also indicate that broader cross-species validation and larger plant-specific training datasets will be required before such models can be generalized across crops [137].

AI-guided protein modeling and generative protein design may also support genome-editor engineering, although their use in plant genome editing remains an emerging direction rather than an established workflow [30,38,39,40,41]. AlphaFold3 extends structure prediction to biomolecular complexes involving proteins, nucleic acids, small molecules and ions, while ProteinMPNN and RFdiffusion support protein sequence design and de novo structure design [38,39,40]. Protein language models further use large-scale sequence information to infer structural features and explore sequence–function relationships [41]. For genome editors, these approaches may help guide future engineering of Cas nucleases, deaminases, reverse transcriptases and linker regions to modify PAM compatibility, editing windows, stability or specificity [9,30,38]. Recent functional validation in rice has provided direct evidence for the applicability of AI-designed nucleases in crop genome engineering. A monocot-optimized OpenCRISPR-1 system enabled efficient single and multiplex editing of *OsSWEET* genes, with mutation frequencies reaching 100% at some targets in T_0_ plants; triple-knockout lines also exhibited broad-spectrum resistance to multiple strains of *Xanthomonas oryzae pv. oryzae*. Moreover, the OpCas9-based prime editor OpenPE6c achieved precise editing efficiencies broadly comparable to those of SpCas9-PE6c in rice protoplasts while producing fewer imprecise editing by-products overall, supporting the functional utility of AI-designed genome-editing proteins in crop systems [138]. However, computationally designed variants must still be validated in plant cells, regenerable tissues and elite crop genotypes before their value can be established [9,30].

For target discovery, AI is best regarded as a prioritization tool rather than a substitute for experimental validation [29,30,35]. Pangenome resources, population variation, QTL and GWAS signals, multi-omics profiles and high-throughput phenotyping can jointly refine candidate genes and regulatory elements for editing, but their causal roles still require testing [29,30,35,114]. Field phenotyping platforms, including UAV imaging, hyperspectral sensing and root phenotyping, can generate quantitative trait data at scales difficult to achieve by manual scoring [35]. These datasets are most valuable when linked to multi-environment trials, because edited alleles must be evaluated against genotype-by-environment variation in target environments [35]. AI-assisted workflows should therefore be used to select candidates, interpret high-dimensional phenotypes and guide subsequent rounds of target or allele design, rather than replace field testing [30,35]. Their reliability will depend on data quality, standardized phenotyping, cross-environment sampling and independent genetic and agronomic validation [30,35,114]. The increasing availability of genomic, molecular and phenotypic data is also changing how candidate edits are prioritized and evaluated. Pangenomic variation broadens the range of accessible alleles and sequence contexts, while multi-omic evidence can help place candidate loci within relevant regulatory and physiological networks [11,12,29]. AI-assisted prediction may further narrow the design space before experimental testing by integrating sequence features and expected editing outcomes [30,36,37]. The resulting edited alleles can then be evaluated through high-throughput phenotyping and multi-environment trials, providing information on phenotypic stability and environmental dependence [30,35]. These observations can, in turn, refine subsequent target selection and allele design, linking computational prioritization with experimental and agronomic validation in an iterative manner [11,12,29,30,35].

## 6. Breeding Validation and Regulatory Considerations

Molecular confirmation of the intended edit is only the first step in evaluating breeding value. Stable inheritance and whole-plant phenotyping should be followed, where appropriate, by replicated field trials and multi-environment evaluation, particularly for quantitative and stress-responsive traits whose effects can vary with genetic background and environment [2,3,29,35,101,102,103]. Field assessment should consider the target trait together with yield, development, product quality and other potential trade-offs, because a molecularly precise edit may not retain agronomic value across genetic backgrounds or environments.

Regulatory assessment adds a further consideration for the deployment of genome-edited germplasm and can differ according to the molecular outcome, the presence of residual foreign DNA and the jurisdiction in which the edited material is evaluated [139]. Site-directed nuclease (SDN) frameworks commonly distinguish SDN-1 changes generated without a repair template, SDN-2 modifications involving short repair templates and SDN-3 events involving larger DNA introduction [139]. Base and prime editing can generate defined sequence changes without conventional DSB-mediated repair, and their regulatory treatment therefore does not always correspond directly to DSB-centered SDN categories. Delivery history and final foreign-DNA status provide complementary information for event characterization rather than serving as stand-alone determinants of regulatory status [84,85,86,139].

Regulatory approaches to genome-edited crops differ substantially among jurisdictions and continue to evolve. In the United States, the judicial vacatur of the 2020 SECURE rule returned USDA-APHIS oversight to the pre-2020 regulatory framework, while earlier regulatory decisions issued under the vacated rule remained valid [140]. China has introduced a differentiated safety-evaluation framework for agricultural gene-edited plants, with regulatory requirements depending on the characteristics and risk profile of the resulting product [141]. In Japan, certain genome-edited products without introduced foreign DNA can fall outside conventional GMO regulation, although prior consultation and notification procedures are used for genome-edited foods [142]. More recently, the European Union adopted Regulation (EU) 2026/1388, establishing a dedicated framework for plants obtained by certain new genomic techniques and distinguishing categories of NGT plants according to the nature of the genetic modification [143].

## 7. Conclusions

Crop genome editing has progressed from targeted mutagenesis to precise modification of nucleotide sequences, gene regulation, genome structure and multiple loci. Nuclease-mediated editing remains effective for generating loss-of-function alleles, whereas base editing, prime editing and *cis*-regulatory editing expand the capacity to create sequence-defined and quantitative allelic variation. The phenotypic effects of edited alleles are shaped by trait architecture, genetic background, gene dosage and environmental conditions, and their breeding value ultimately depends on stable inheritance and reproducible agronomic performance.

Advances in delivery and regeneration are broadening the range of crop genotypes that can be edited, while pangenomics, multi-omics and high-throughput phenomics are improving the identification and evaluation of breeding-relevant targets. Artificial intelligence is increasingly extending these capabilities by supporting guide-RNA design, prediction of editing outcomes, prioritization of candidate genes and alleles, and the engineering of genome-editing proteins. Recent crop studies also indicate that AI-designed editors can function in plant genome-editing systems, although their performance remains dependent on target locus, crop genotype and experimental context. The integration of AI with genomic, molecular and phenotypic information may therefore improve the efficiency and predictability of allele design, but experimental and field validation remain essential for complex agronomic traits.

Genotype-dependent transformation and regeneration, polyploid genome complexity, unintended genomic changes and variation in performance across environments continue to limit the translation of precise molecular editing into stable crop improvement. Increasing integration of genome editing with genetic diversity, molecular information, predictive modeling and phenotypic evaluation is expected to support more precise and adaptable breeding strategies for complex traits and diverse production environments.

## Data Availability

This manuscript did not involve the generation of new data. All data and information discussed herein are based on previously published literature cited in the article and listed in the References Section.
